# Impact of the COVID-19 pandemic on computational biology early career researchers: A global retrospective study

**DOI:** 10.1371/journal.pcbi.1013554

**Published:** 2025-10-15

**Authors:** Pradeep Eranti, Megha Hegde, Syed Muktadir Al Sium, R. Gonzalo Parra, Alastair M. Kilpatrick, Sayane Shome, Farzana Rahman

**Affiliations:** 1 Université Paris Cité, Inserm, T3S, Paris, France; 2 School of Computing and Mathematics, Faculty of Engineering, Computing, and the Environment, Kingston University London, London, United Kingdom; 3 Industrial Microbiology Research Division, BCSIR Chattogram Laboratories, Bangladesh Council of Scientific and Industrial Research (BCSIR), Chattogram, Bangladesh; 4 Computational Biology Group, Life Sciences Department, Barcelona Supercomputing Center, Barcelona, Spain; 5 Centre for Regenerative Medicine, Institute for Regeneration and Repair, The University of Edinburgh, 4-5 Little France Drive, Edinburgh BioQuarter, Edinburgh, United Kingdom; 6 Department of Anesthesia, Perioperative, and Pain Medicine, Stanford University School of Medicine, Stanford, California, United States of America; Montreal, CANADA

## Abstract

The COVID-19 pandemic led to devastating physical, psychological, and financial impacts on millions of people across the world. Amidst a rapidly evolving research landscape, the global scientific community was forced to swiftly adapt to novel working methods, including remote collaboration tools, virtual conferences, and online research platforms. Surveys of life sciences researchers have indicated that computational biologists experienced less disruption and a smoother transition to remote working than experimental biologists, due to their reduced reliance on laboratory equipment. Despite this adaptability, the sudden shift to remote work, compounded by stress and social isolation, has posed significant mental health challenges for these workers. However, remote work has also facilitated opportunities for more flexible work arrangements and increased collaboration across geographical boundaries. To investigate these impacts, we conducted surveys of computational biologists during the Intelligent Systems for Molecular Biology (ISMB) conferences in 2020 and 2021, which were held virtually due to the COVID-19 lockdowns. This study implements a thorough statistical analysis of the survey results to offer insights into the repercussions of the lockdowns on researchers and their work. Key areas of investigation include the effects of institutional support (or lack thereof), the difference in productivity compared to pre-lockdown levels, and the significance of gender in determining these impacts. Notably, a lack of institutional support with regard to mental health and finances was shown to have a significant negative effect on early-career researchers. Although limited by a small sample size, our study sets the stage for a more robust exploration of these trends in future research. Importantly, by illuminating the challenges and opportunities arising from the COVID-19 pandemic and lockdowns, our study offers hope for potential solutions supporting the well-being of early-career researchers in unprecedented circumstances.

## Introduction

The COVID-19 pandemic has caused unprecedented disruption to every aspect of human life, significantly impacting individuals’ physical, mental, and financial well-being worldwide [1]. The scientific community is no exception, with the pandemic forcing researchers to adapt quickly to new working methods and navigate a swiftly evolving research landscape [2].

The pandemic has impacted all scientific fields [3]; data from surveys specific to the life sciences have suggested that researchers working in the fields of computational biology and bioinformatics were relatively unaffected by the disruption caused by the pandemic [4] (here, we describe researchers in these two fields and associated disciplines under the umbrella term “computational biology researchers” throughout). Proposed reasons for this include less disruption to ongoing research projects because of institutional closures, a decreased reliance on laboratory equipment, and an increased technical knowledge, allowing a more accessible adaptation to new remote working technologies.

As in other fields, computational biology researchers had to quickly adapt to remote work arrangements. While a “work-from-home” culture is now relatively standard, this was a rare option pre-pandemic. Although computational biology relies heavily on “dry lab” research including, but not limited to, applied data analysis, algorithm development, and computer simulations, the adjustment to working remotely and collaborating online with peers can be challenging when working on collaborative projects that require close teamwork.

This transition to remote work, coupled with the stress and uncertainty of the pandemic, has placed a considerable burden on many individuals’ mental health and well-being, leading to newly defined health conditions such as “Zoom fatigue” [5]. Here, we aim to explore the impact of the COVID-19 pandemic on computational biology early-career researchers (ECRs) through analysis of data collected via online surveys in 2020 and 2021. Throughout this paper, ECRs are referred to as researchers (i) doing research (Master researcher or PhD student) under the supervision of an experienced independent researcher and (ii) researchers who completed PhD or equivalent level of competence and experience but are not yet established as an independent researcher developing their research scope, attracting funding or leading a research group [6].

Although the first signs of the COVID-19 pandemic were observed in December 2019, several governments worldwide started implementing stringent measures in March 2020, including mandated lockdowns promoting social distancing and mask-wearing to combat the spread of the COVID-19 virus. Lockdowns continued until late 2020 when some social isolation measures were slightly relaxed; however, a second deadlier wave of the COVID-19 virus became prominent between January and March 2021, leading to stricter lockdowns observed by various countries worldwide. As part of the lockdowns in 2020 and 2021, many universities and research institutions wholly or partially shut down, drastically reducing in-person academic work [4,7] and forcing a rapid shift to online delivery of computational biology training [8]. The majority of the survey data capture responses from the initial lockdown period of Summer 2020, with the 2021 dataset providing additional context a year further into the pandemic.

The survey questionnaires were distributed via virtual conferences and social media platforms with a focus on individuals working in computational biology worldwide. The surveys aimed to gather data regarding the experiences of ECRs in the field during the pandemic. Conducting surveys is a valuable approach to comprehend the effects of the pandemic on individuals within specific domains, including computational biology [9,10].

In this study, we aim to provide comprehensive data-based insights on how the COVID-19 pandemic has affected ECRs in computational biology. By shedding light on the challenges and opportunities presented by the pandemic, we aim to provide a foundation for forthcoming research and policies to support the well-being of individuals working in this pivotal field as the world moves beyond the pandemic where frequent remote work and virtual/hybrid meetings/conferences seem to be the new normal.

## Methods

### Ethics statement

This study received ethical approval from the International Society for Computational Biology (ISCB) Student Council Executive Team (2018–2021), who oversees the ethical approval and standard of survey, process and protocols. The survey protocol was reviewed to ensure adherence to established ethical standards.

Participation was entirely voluntary. Informed written consent was obtained electronically from all respondents via a mandatory checkbox at the start of the survey. Respondents were clearly informed that their participation was anonymous and that their responses, including any free-text comments, could be used for analysis and shared in public forums in anonymised form by the ISCB Student Council and the survey authors. Participants had full right to withdraw at any stage of the survey without further consequences.

The survey did not collect any personally identifiable information. All data were handled in aggregate to preserve participant confidentiality. No individuals under the age of 18 were included in the study, and thus parental consent was not required.

### Data collection

We developed a survey to understand how the COVID-19 pandemic impacted the work and lives of computational biology ECRs. Although the main focus of the study was ECRs, we also collected responses from senior researchers and industry professionals in the field. The survey questionnaire was distributed via email among participants of the virtually held joint Intelligent Systems for Molecular Biology (ISMB) and European Conference on Computational Biology (ECCB) conference in July 2020 and the virtual ISMB conference in July 2021. Identical surveys were used in 2020 and 2021. The majority of responses (77.1% in 2020 and 90.7% in 2021) were obtained within five days of the conferences.

The survey consisted of 31 questions, which could be answered by multiple-choice options or by text input if respondents felt no suitable choice was available. An additional freeform section allowed responders to comment on issues not raised by the survey questions. Survey questions were worded to consider several factors regarding the exact career stage, workload, transition to remote working, and institutional support experienced by ECRs, as well as questions regarding respondents’ living situation and mental health. The survey questionnaire was reviewed by members of the International Society for Computational Biology’s (ISCB) Student Council (SC) and followed ISCB’s General Data Protection Regulation (GDPR) and ethical regulations. Once the survey was formulated, the survey link was distributed to ISMB/ECCB participants via email by the conference organizing teams. A link to the survey was also distributed via X (previously known as Twitter) and Facebook. The ISCB-SC executive team members, at that time, were involved in the data collection process; the authors conducted data processing and analysis presented in this study.

**Fig 1:**
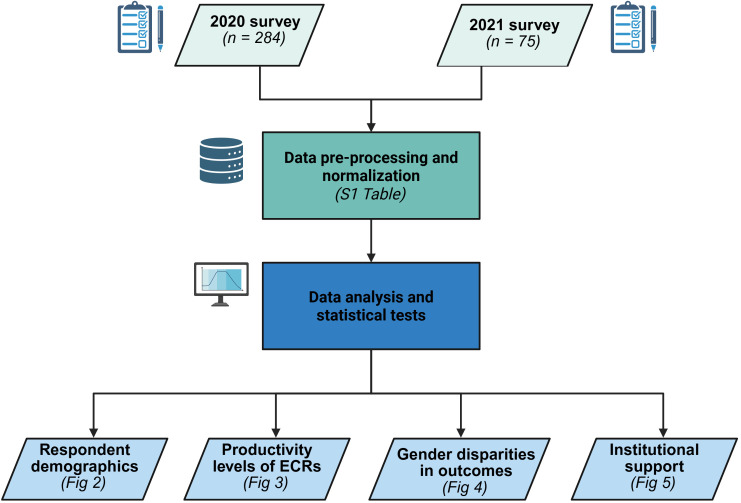
Workflow represents the process followed throughout the study, from data collection to analysis. This figure is created using biorender (https://www.biorender.com/). The icons used from https://fontawesome.com/icons (only the free icons are used) and licensing details are given here: https://fontawesome.com/v4/license/.

The response data (2020 n=284; 2021 n=75) was collected through Google Forms and exported in Microsoft Excel format for subsequent preprocessing, cleaning, and analysis. While we collected demographic data about the respondents, responses were otherwise anonymous. We obtained consent from survey participants to use their responses in this study and publish the data anonymously. The raw data used in this study are available in the Zenodo repository [11]. The overall workflow is summarized in Fig 1, which represents the process followed throughout the study, from data collection to analysis.

In addition, we considered the conference registration data from the 2020 and 2021 ISMB conferences and the 2021–22 Annual Report of the ISCB Equity, Diversity, and Inclusion (EDI) Committee [12] as references for testing the validity and generalizability of the survey results.

### Data preprocessing

The survey data was imported into R (v.4.2.3) using the readxl (v.1.3.0) tidyverse package [13]. Data from the textual questions were processed to group responses with similar meanings (for instance, phrases., “I don’t know,” “Don’t know,” and “Do not know” were merged into a singular response of “Don’t know” for question 18). Any ungrouped text responses were classified as ‘Other.’ For question 7 (S1 Table), which asked about productivity levels compared to pre-COVID, all answers quoting figures above 100% were grouped into a single category of “>100”.

These preprocessing steps were carried out using the mutate function in the dplyr R package (v.1.1.2) [14]. The S1 File provides additional information on data preprocessing.

### Data analysis and statistical models employed

Statistical analyses were performed using the stats package in R [15], using Chi-squared tests (with post-hoc tests as appropriate), Fisher’s Exact tests, and Welch two-sample t-tests, as appropriate. For Fisher’s Exact tests with contingency tables larger than 2x2, p-values were computed by Monte Carlo simulation with 2x10^5^ replicates. The threshold for statistical significance was set at *p* < 0.05. Analyses based on linear models used a binomial generalized linear model using the glm function in R. Analysis of timestamp data was conducted using the lubridate R package [16]. Data visualization was performed with R and Python (v.3.10.11) using the Pandas [17], Matplotlib [18], and Seaborn [19] packages.

## Results

### Respondent demographics

To obtain an understanding if the survey responses are reflective of the general population of computational biology researchers, we compared the respondent demographic data from the two surveys with data on ISCB membership (n = 3,208) from the 2021–22 Annual Report of the ISCB Equity, Diversity and Inclusion (EDI) Committee as of June 2022 [19], and registration data from the 2020 and 2021 ISMB conferences. The survey respondent’s data represents 16.0% (*n* = 284/1,775) and 3.5% (*n* = 75/2,116) of registrants to these conferences, respectively.

**Fig 2:**
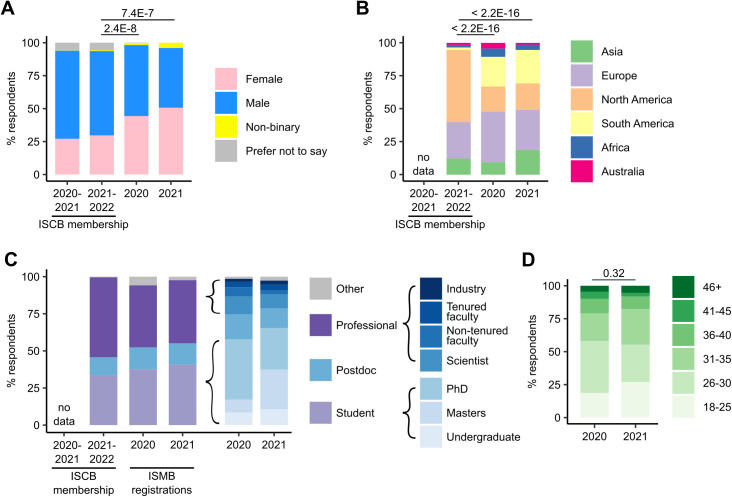
Stacked barplots representing the proportions of survey responses. These are compared to ISCB membership data where available. **A.** Respondent genders. **B.** Respondent locations (continent). **C.** Respondent career stages. These are also compared to registration data for the corresponding ISMB conferences. **D.** Respondent ages.

For the survey questions on gender, location, and career stage of respondents, we had a 100% response rate (Fig 2A). 44.4% (*n* = 126/284) of respondents in 2020 were female; 53.9% (*n* = 153/284) were male. In 2021, the proportion of female respondents increased to 50.7% (*n* = 38/75); 45.3% (*n* = 34/75) of respondents were male. The equivalent question on gender in the ISCB EDI 2021–2022 report had a response rate of 70.7% (*n* = 2,267/3,208). In both 2020 and 2021, the proportion of female respondents to the survey was significantly higher than the proportion of female ISCB members (*n* = 673/2,267 [29.7%]; Chi-squared tests, 2020 *χ*^2^ = 38.4, df = 3, *p* = 2.4E−8; 2021 *χ*^2^ = 31.3, df = 3, *p* = 7.4E−7). 1.4% of respondents in 2020 (*n* = 4/284) and 4.0% (*n* = 3/75) in 2021 identified as non-binary. This proportion is higher than in overall ISCB members (*n* = 13/2,267 [0.6%]); however, due to low statistical power, it is not possible to say whether the responses from non-binary ECRs reflect wider trends.

The equivalent question about the respondent’s location in the ISCB EDI report (2021–2022) had no reported response rate (*n* = 3,221) but included details about the geographical representation of the ISCB members (Fig 2B). The majority of respondents to the survey (38.4% [*n* = 109/284] in 2020 and 30.7% [*n* = 23/75] in 2021) were from Europe rather than North America as in the ISCB membership (*n* = 1,764/3,221). We observe significantly different proportions of geographical representation of respondents in both years 2020 and 2021 (Chi-squared tests, 2020 *χ*^2^ = 421.2, df = 5, *p* < 2.2E−16; 2021 *χ*^2^ = 186.5, df = 5, *p* < 2.2E−16), and the proportion of respondents from Europe was significantly higher in the 2020 data (Chi-squared post-hoc test, *p* = 1.3E−3).

Most of the survey respondents comprise PhD students and postdoctoral researchers (40.5% [*n* = 115/284] and 16.9% [*n* = 48/284], respectively, in 2020 and 28.0% [*n* = 21/75] and 13.3% [*n* = 10/75] in 2021) (Fig 2C). ISCB membership data (*n* = 3,208; 100% response rate) does not allow analysis at a finer resolution (categories: “Student,” “Postdoc, ” “Professional, ” “Other”). However, students make up 33.4% [*n* = 1,073/3,208] of ISCB membership, while undergraduate, masters and PhD students make up significantly larger proportions of our 2020 (57.8%; *n* = 24/284, 25/284, 115/284, respectively) and 2021 (65.3%; *n* = 8/75, 20/75, 21/75, respectively) survey responses (Chi-squared test, 2020 *χ*^2^ = 66.3, df = 1, *p* = 3.9E−16; 2021 *χ*^2^ = 31.7, df = 1, *p* = 1.8E−8). We also find significantly higher proportions of students (undergraduate, masters, and PhD) in our 2020 and 2021 data than in the registrations of the corresponding ISMB conferences (2020 37.6%, *n* = 667/1,775; 2021 40.6%; *n* = 860/2,116) (Chi-squared test, 2020 *χ*^2^ = 40.5, df = 1, *p* = 1.9E−10; 2021 *χ*^2^ = 17.2, df = 1, *p* = 3.4E−5). 12.3% (*n* = 393/3,208) of ISCB members are postdocs, while postdocs make up significantly larger proportions of 2020 (*n* = 48/248; 19.4%) and 2021 (*n* = 10/75; 13.3%) data (Chi-squared test, 2020 *χ*^2^ = 4.7, df = 1, *p* = 3.0E−2; 2021 *χ*^2^ = 12.5, df = 1, *p* = 4.0E−4). However, the proportions of postdocs in the data were not significantly different from those in the registrations of the corresponding ISMB conferences (2020 14.8%, *n* = 262/1,775; 2021 14.5%, *n* = 307/2,116) (Chi-squared test, 2020 *χ*^2^ = 0.7, df = 1, *p* = 4.0E−1; 2021 *χ*^2^ = 1.3E−2, df = 1, *p* = 9.1E−1).

Together, these data reflect the focus on ECRs in this study. Accordingly, responses to the question on age (Q2; S1 Table, response rate in 2020, 2021 is 99.6% and 100% respectively) revealed a large majority of respondents (78.5% [*n* = 223/283] in 2020; 81.3% [*n* = 61/75] in 2021) were aged 18–35 (Fig 2D). We found no significant differences in the proportions of age groups between the 2020 and 2021 data (Chi-squared test, *χ*^2^ = 5.9, df = 5, *p* = 3.2E−1).

### Impact of COVID-19 on productivity levels of computational biology ECRs

**Fig 3:**
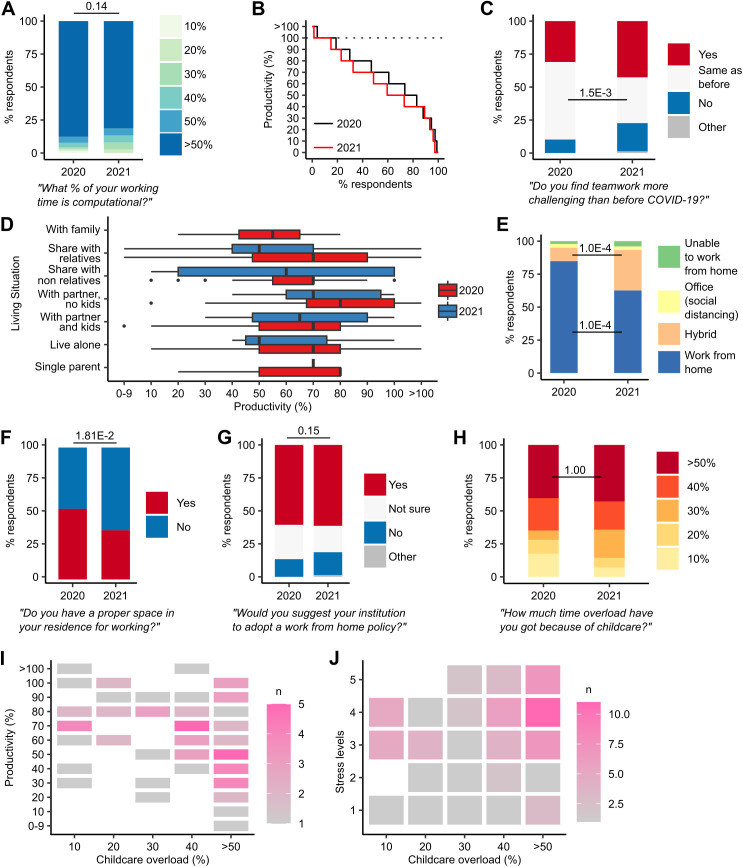
Visualisation representing proportions of survey responses. A. Stacked barplot representing the proportion of respondent time spent on computational work. **B.** Step plot of productivity against the percentage of survey respondents. **C.** Stacked barplot representing responses to the survey question on teamwork. **D.** Boxplots summarizing responses to living situations. **E.** Stacked barplot representing responses to work setup. **F.** Stacked barplot representing respondent home working setups. **G.** Stacked barplot summarizing respondent views on institutional working-from-home policies. **H.** Stacked barplot representing respondent time overload due to childcare responsibilities. **I.** Heatmap summarizing data on respondent time overload due to childcare and respondent productivity. **J.** Heatmap summarizing data on respondent time overload due to childcare and respondent stress levels.

Based on responses to Q23 (S1 Table), we found that 87.7% (*n* = 249/284) of respondents in 2020 spent the majority of their time carrying out computational work. This proportion was slightly lower in 2021, at 81.3% (*n* = 61/75), though not significantly (Chi-squared test, *χ*^2^ = 8.3, df = 5, *p* = 0.14) (Fig 3A).

Responses to the question on productivity (Q7; S1 Table), 100% response rate in 2020 and 2021 showed that, in 2020, 81.1% of computational biology ECRs who gave a numerical response (*n* = 223/275) rated their productivity as having decreased compared to pre-pandemic, with 14.9% (*n* = 41/275) reporting no change and 4.0% (*n* = 11/275) rating their productivity as increased. In 2021, 85.1% of respondents who gave a numerical response (*n* = 63/74) rated their productivity as having decreased, with 13.5% (*n* = 10/75) reporting no change and 1.4% (*n* = 1/74) rating their productivity as increased (Fig 3B). The mean productivity rate was 68.7% in 2020 (female 68.3%; male 68.8%) and 63.9% in 2021 (female 64.0%; male 63.2%). Of respondents reporting a decrease in their productivity, 32.7% in 2020 (*n* = 73/223) and 47.6% in 2021 (*n* = 30/63) reported a strong impact (≤50%) on their productivity, compared to pre-pandemic. We found no statistically significant association between ECR career stage and productivity in either the 2020 or 2021 data (Chi-squared tests, *χ*^2^ = 84.8, *p* = 0.17 and *χ*^2^ = 86.5, *p* = 0.34, respectively).

Responses to the question on teamwork (Q16; S1 Table) showed that in 2020, 31.0% (*n* = 88/284) of ECRs reported that they found teamwork more challenging compared to before the pandemic (Fig 3C). 58.8% of respondents (*n* = 167/284) found teamwork to be similarly challenging to before the pandemic. In 2021, the proportion of respondents who found teamwork similarly challenging had decreased significantly to 34.7% (*n* = 26/75) (Chi-squared post-hoc test, *p* = 1.5E−3); however, while the proportion of respondents who found teamwork more challenging had increased to 42.7% (*n* = 32/75), this increase was not significant (Chi-squared post-hoc test, *p* = 0.45).

Analysis of the 2020 data on ECR living conditions (Q25; S1 Table) reveals a significant association between living conditions and productivity (Fisher’s exact test, *p* = 0.02) (Fig 3D). We did not find a similarly significant association in the 2021 data (*p* = 0.35). Further, we find a significant association between living conditions and a strong impact on productivity in the 2020 data (Fisher’s Exact test, *p* = 7.0E−3).

Responses to our data on respondent working conditions (Q6; S1 Table) indicated that the majority of respondents in 2020 (84.9%, *n* = 241/284) worked from home during lockdown periods. In the 2021 data, this proportion was significantly lower, at 62.7% (*n* = 47/75) (Chi-squared post-hoc test, *p* = 1.0E−4); the proportion of respondents’ hybrid working increased significantly, from 10.2% (*n* = 29/284) in 2020, to 30.7% (*n* = 23/75) in 2021 (Chi-squared post-hoc test, *p* = 1.0E−4) (Fig 3E).

Responses to Q28 (S1 Table) indicated that in 2020, 53.5% (*n* = 152/284) of ECRs had a dedicated space for working at home. In 2021, this proportion had significantly decreased to 37.3% (*n* = 28/75) (Chi-squared test, *χ*^2^ = 5.6, df = 1 *p* = 1.81E−2). Among ECRs working at least in part from home, 47.1% (*n* = 115/248) reported that they had a dedicated working space in 2020. These respondents reported higher productivity levels (mean 71.7%, sd 21.5) than those without a dedicated working space and instead working in a shared space (e.g., living room, kitchen, bedroom) (*n* = 133/248; mean 68.0%, sd 23.6). In 2021, respondents with a dedicated working space (34.9%; *n* = 23/66) reported lower productivity (mean 64.8%, sd 22.9) compared to those working in a shared space (65.1%; *n* = 43/66; mean 66.5%, sd 22.8) (Fig 3F).

Responses to Q8 (S1 Table) indicated that the majority of respondents (60.6%; *n* = 172/284) would suggest transitioning to working-from-home model post-COVID; 13.4% (*n* = 38/284) of respondents would not suggest such a model. These proportions were not significantly different in 2021, where 61.3% (*n* = 46/75) of respondents would suggest a working-from-home model, while 17.3% (*n* = 13/75) would not (Chi-squared test, *χ*^2^ = 5.3, df = 3, *p* = 0.15) (Fig 3G).

Survey Q26 and Q27 (S1 Table) enquired as to how much “time overload” respondents felt. Here, we define “time overload” as how much extra burden respondents felt they had due to childcare (Q26) and domestic responsibilities (Q27) during the pandemic. 16.9% of responders in 2020 (total *n* = 48/284; “I live with partner with kids” *n* = 45/284; “I am a single parent” *n* = 3/284) and 17.3% of responders in 2021 (total *n* = 13/75; “I live with partner with kids” *n* = 12/75; “I am a single parent” *n* = 1/75) had childcare responsibilities (Q25; S1 Table). Response rates to our question on time overload due to childcare were 20.0% (*n* = 57/284) and 18.7% (*n* = 14/75) in 2020 and 2021, respectively. Asked how much time overload they felt due to childcare, 40.4% (*n* = 23/57) of respondents in 2020 chose the highest option, “More than 50%.” This proportion was higher in 2021 (42.9%; *n* = 6/14), although not significantly so (Chi-squared test, *χ*^2^ = 2.29E−31, df = 1, *p* = 1.00) (Fig 3H). Respondents with greater time overload due to child-care responsibilities reported lower productivity levels and higher stress levels than those without such responsibilities (Fig 3I and 3J).

Together, the data show that various aspects of the COVID-19 pandemic negatively impacted productivity among computational biology ECRs.

### Impact of COVID-19 on male versus female computational biology ECRs were more severely impacted

**Fig 4:**
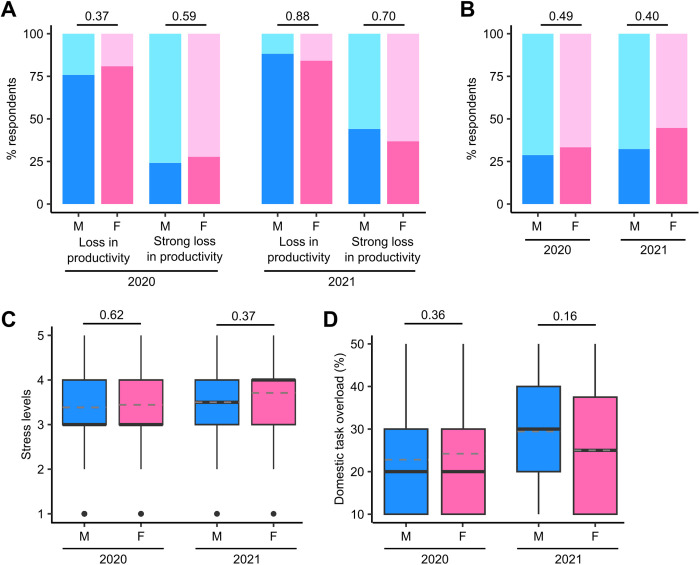
Stacked barplots and boxplots representing proportions of survey responses. A. Stacked barplots representing respondents who indicated a loss or strong loss in productivity, split by gender. **B.** Stacked barplots representing respondents who reported work lost due to COVID-19, split by gender. **C.** Boxplots of respondent stress levels, split by gender. Dashed lines represent group means. **D.** Boxplots of respondents’ time overload due to domestic tasks, split by gender. Dashed lines represent group means.

Data on respondents’ gender (Fig 2A) allows an insight into gender-based effects. Further analysis of responses to Q7; S1 Table (Fig 3B) reveals that in the 2020 data, 75.8% (*n* = 116/153) of male ECRs reported a loss in productivity; the proportion of female ECRs reporting a loss in productivity was higher, although not significantly so (81.0%, *n* = 102/126; Chi-squared test, *χ*^2^ = 0.79, df = 1, *p* = 0.37). Female ECRs were also more likely to report a strong (≤50%) loss in productivity, with 27.8% (*n* = 35/126) of females reporting this, compared to 24.2% (*n* = 37/153) of males, although again, this was not statistically significant (Chi-squared test, *χ*^2^ = 0.30, df = 1, *p* = 0.59). In our 2021 data, lower proportions of female ECRs reported a loss in productivity (84.2%, *n* = 32/38 versus males 88.2%, *n* = 30/34) or strong loss in productivity (36.8%, *n* = 14/38 versus males 44.1%, *n* = 15/34); again these differences were not statistically significant (Chi-squared tests, *χ*^2^ = 0.02, df = 1, *p* = 0.88; *χ*^2^ = 0.15, df = 1, *p* = 0.70) (Fig 4A).

30.6% (*n* = 87/284) of respondents in 2020 reported losing research work in some way due to the pandemic (Q9; S1 Table). While this proportion was higher in the 2021 data, at 37.3% (*n* = 28/75), the increase was not significant (Chi-squared test, *χ*^2^ = 0.93, df = 1, *p* = 0.33). Female ECRs were more likely to have lost research work due to the pandemic (33.3%, *n* = 42/126) compared to males (28.8%, *n* = 44/153) in the 2020 data, although not significantly so (Chi-squared test, *χ*^2^ = 0.5, df = 1, *p* = 0.49). Similarly, in the 2021 data, 44.7% of female ECRs reported lost research work (*n* = 17/38), compared to 32.4% of males (*n* = 11/34) (Chi-squared test, *χ*^2^ = 0.7, df = 1, *p* = 0.40) (Fig 4B).

The question on work-related stress (Q17; S1 Table) revealed a mean stress score of 3.40 (sd = 1.00) in the 2020 data on a scale of 1–5. This increased, although not significantly, to 3.57 (sd = 1.00) in the 2021 data (Welch two-sample *t* test, *t* = −1.3, df = 115.5, *p* = 0.20). In the 2020 data, female respondents reported higher stress levels (mean 3.44, sd 0.96) than their male counterparts (mean 3.39, sd 1.03), although this was not statistically significant (Welch two-sample *t* test, *t* = 0.49, df = 272.6, *p* = 0.62). Similarly, in the 2021 data, stress levels were higher for female respondents (mean 3.71, sd 0.90) than male respondents (mean 3.50, sd 1.05), although not significantly so (Welch two-sample *t* test, *t* = 0.91, df = 65.3, *p* = 0.37) (Fig 4C).

Responses to the question on domestic duties (Q27; S1 Table) revealed a mean time overload score of 2.36 (sd = 1.27) in 2020. This increased, although not significantly, to 2.69 (sd = 1.34) in the 2021 data (Welch two-sample *t* test, *t* = −1.9, df = 112.2, *p* = 5.67E−2). In the 2020 data, female respondents reported a greater time overload (mean score 2.42, sd 1.25) than males (mean 2.28, sd 1.28), although not significantly so (Welch two-sample *t* test, *t* = 0.92, df = 269.2, *p* = 0.36). In 2021, female respondents reported a lower time overload (mean 2.50, sd 1.35) than males (mean 2.94, sd 1.30), although again, this difference was not significant (Welch two-sample *t* test, *t* = −1.41, df = 69.6, *p* = 0.16) (Fig 4D).

Splitting the data on gender inevitably leads to a loss in statistical power due to smaller sample sizes. However, these observations are suggestive of an increased impact of the COVID-19 pandemic on female ECRs in computational biology.

### A lack of institutional support affected computational biology ECRs

**Fig 5:**
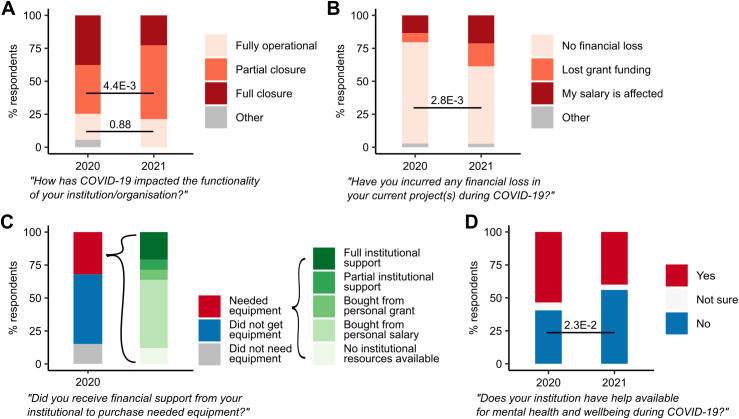
Stacked barplots representing proportions of survey responses. **A.** Impact of COVID-19 on organization functionality. **B.** Financial losses in research projects due to COVID-19. **C.** Availability of institutional financial support. **D.** Availability of institutional mental health resources.

Responses to the question on workplace functionality and closures (Q5; S1 Table) indicate that 74.6% (*n* = 212/284) of respondent’s institutions were at least partially closed (<50% operational) in July 2020, with 50.5% of these (*n* = 107/212) full closures (<5% operational) (Fig 5A). The proportion of unaffected respondent institutions had not changed significantly by July 2021 (2020: 19.7%, *n* = 56/284; 2021: 21.3%, *n* = 16/75; Chi-squared test, *χ*^2^ = 2.2E−2, df = 1, *p* = 0.88). However, the proportion of partial closures had significantly increased, from 37.0% (*n* = 105/284) to 56.0% (*n* = 42/75) (Chi-squared test, *χ*^2^ = 8.1, df = 1, *p* = 4.39E−3), suggesting a trend to return to pre-pandemic levels of operation.

Responses to the question on financial losses to research projects (Q11; S1 Table) revealed that by July 2020, 7.0% (*n* = 20/284) of respondents had suffered a loss of grant funding (Fig 5B). A further 13.4% (*n* = 38/284) responded that their personal salary had been affected in some other way. The proportion of respondents reporting no financial losses to their projects significantly decreased from 76.8% (*n* = 218/284) in 2020 to 58.7% (*n* = 44/75) in 2021 (Chi-squared test, *χ*^2^ = 9.0, df = 1, *p* = 2.77E−3).

Responses to our question on institutional financial support (Q31; S1 Table) revealed that 15.1% of ECRs in 2020 (*n* = 43/284) did not require any additional equipment for home working. The majority of respondents (52.8%; *n* = 150/284) reported that they did not get any equipment, although it is unclear whether they did not require equipment or equipment was requested but not provided. Of the remaining respondents who chose a more detailed answer (*n* = 91), 63.7% (*n* = 58/91) either reported that there was no institutional financial support available or bought required equipment from their own personal salary. Twenty-six respondents received either partial or full financial support from their institution to buy the required equipment (Fig 5C).

More than 40.5% of respondents in the 2020 data (*n* = 115/284) reported that their institution had no support available for staff or student mental health and wellbeing during the pandemic (**Q18;** S1 Table). This proportion increased significantly to 56.0% (*n* = 42/75) in the 2021 data (Chi-squared test, *χ*^2^ = 5.2, df = 1, *p* = 2.28E−2). (Fig 5D)

## Discussion

Even before the COVID-19 pandemic, the traditional work paradigm for dry-lab computational biologists was largely centered around on-site operations, with people commuting to physical offices and meetings conducted face-to-face. The concept of remote work and virtual conferences was often met with skepticism and resistance, with a lack of appropriate institutional policies, with concerns about productivity, collaboration, and maintaining a professional environment from home [20,21]. However, the pandemic catalyzed a seismic shift in these perceptions. It necessitated the exploration of alternative work arrangements and demonstrated that remote work and virtual meetings could be viable and equitable options. The forced experiment of working from home revealed that productivity could be maintained, if not enhanced, and that virtual platforms could facilitate effective communication and collaboration [20,21]. This shift has opened up new possibilities for work-life balance, inclusivity, and flexibility, challenging the traditional norms of the workplace. Further, major conferences in computational biology (such as Intelligent Systems in Molecular Biology and computer science more generally (such as Computer Vision and Pattern Recognition and Neural Information Processing Systems) now routinely offer hybrid options so people worldwide can attend, regardless of their location. However, the transition to home working during the pandemic, coupled with many other factors, undoubtedly had an effect, mostly negative, on early-career computational biologists.

Our data shows a higher proportion of ECRs from South America than expected. We speculate that this is partially attributable to the work of ISCB Regional Student Groups in this region [22,23]; however, we do not discount potential sample bias. While we have demographic data for survey respondents at a regional level, we have no data to make conclusions at the country level. The variability in regional impacts of the COVID-19 pandemic has been discussed previously [24] and is tied to governmental responses, for example, in terms of the severity and lengths of lockdowns and other restrictions and vaccination rollouts. We do, however, note that to make meaningful conclusions at a country level would require a much larger sample size. In addition, the data collected in this study does not permit us to examine other location-based factors. For instance, we expect that a respondents’ region will have an effect on their institution’s ability to support them financially, but small sample sizes preclude making any such conclusions. Similarly, we cannot discern whether the respondents’ affiliated institutions are in urban or rural areas, nor can we determine the economic status of the country where the respondents reside. The reopening process of their affiliated institutions and any associated shifts in behavior or lifestyle are also beyond the scope of this data. While we cannot draw any conclusions based on ethnicity data, this is likely to be a factor in the health of ECRs and their families [25].

The disruption caused by the COVID-19 pandemic affected different scientific fields differently, with bench sciences such as biology and biochemistry previously reporting a 30-40% decline in research time compared to pre-pandemic [3]. Contrary to previous studies [4], we find that productivity in computational biology ECRs was notably impacted, with mean productivity decreased by around a third, consistent with the impact on other life sciences. This decrease was independent of the career stage. 88% of ECRs in the 2020 data spent the majority of their time (≥50%) as “dry lab” scientists. Although such scientists undoubtedly have some advantages in being able to work remotely with greater ease, it is not unusual for dry lab scientists to collaborate closely with bench scientists, who were more directly affected by institutional closures. In this context, some decrease in productivity due to the COVID-19 pandemic seems unavoidable, although, in free text responses (Q21), respondents mentioned factors such as social isolation and a lack of work/life separation as also negatively impacting their productivity.

We find aspects of an ECR’s living situation to have an impact on their productivity. These aspects include who they share a home with, whether they have a dedicated working space, and whether they have additional childcare roles. There are likely to be strong links between each of these factors, particularly for ECRs who may reside in an unfamiliar country, may not have their own home, and may be considering starting a family. The links between these factors are likely complex and not captured completely by the survey responses. For instance, it is feasible that researchers living on their own are more likely to have a dedicated working space. However, any productivity boost associated with this may be negated by the anxiety of social isolation. We suggest that sociocultural factors are also likely to be involved. For instance, in some parts of the world, young people may traditionally live with their family until they are older. The impact of these factors, which may be linked to geographical location and ethnicity, is beyond the scope of the data.

The data suggests that at least 20% of computational biology ECRs were directly affected financially by the early stages of the COVID-19 pandemic. This proportion is likely higher when considering the lack of institutional financial support found in the survey data. The data regarding the financial burden of missed grants in computational biology due to the COVID-19 pandemic mirrors findings across the life sciences more generally. A recent study indicated a marked reduction in financial support for clinical trials, publications, and research projects unrelated to COVID-19 [26]. These financial worries for computational biology ECRs compound more general economic anxieties, which have been observed even among households not directly affected by COVID-19 [27].

The mental health burden of COVID-19 is well-established, especially amongst ECRs, who may have experienced heavier workloads, job insecurity, and geographic instability [28]. As noted above, a large proportion of respondents in 2020 reported that their institution had no mental health support available. Of the respondents who reported that there was support available, further examination of free text responses (Q18) revealed that in some cases, this was only available in theory or self-help resources considered to be of little practical use. While a more thorough investigation of this finding (for example, using psychological metrics such as the Perceived Stress Scale) is beyond the scope of this study, the findings suggest a persistent and disproportionate impact that was worsening as the pandemic continued in 2021.

We acknowledge the limitations of this study, importantly, the sample size and response distribution. Future research should aim to include larger and more diverse datasets to strengthen the generalizability of the findings. For example, while our data suggest more female ECRs suffered lost productivity due to the COVID-19 pandemic, consistent with previous studies [29–31], this was not statistically significant due to the small sample size. While the proportion of female respondents increased in 2021, we note both the smaller sample size and previous studies highlighting higher response rates to online surveys among female faculty compared to male faculty [32].

Incorporating variables such as job responsibilities, access to resources, and support systems would also provide a more comprehensive understanding of the observed patterns. We cannot ascertain if the respondents have the backing of family members or other forms of personal support. While not addressed in the current study, these elements could significantly impact the findings and should be considered in future research endeavors. One potential limitation of the findings is the reliability of self-reported productivity data, which is known to be subjective and susceptible to cognitive biases [24]. However, given the diversity of the cohort and their research, self-reported data may be more accurate than traditional measures of productivity. Finally, while we designed the surveys to be anonymous for individual privacy reasons, it follows that the data between 2020 and 2021 cannot be matched, so it is not possible to see how experiences changed between these time points at an individual level.

Our survey findings highlight a multitude of factors affecting computational biology ECRs during the COVID-19 pandemic. By recognizing and addressing these factors, organizations can create a more resilient and supportive work environment for researchers in these fields moving forward.

## Supporting information

S1 Table Descriptive statistics of responses to the Survey data in 2020 and 2021. There were 284 and 75 responses in the 2020 and 2021 datasets, respectively. If there is a change in the number of responses (N), “N” is marked accordingly next to the question. The questions compulsory to answer are marked with “*” in the first column: Q1–9, Q11–13, Q16–18, Q20–25, and Q27–31. Respondents have the option to select only one choice (Q1–14, Q16–18, Q20, Q23–29, Q31) and, to some questions, have the option to select multiple choices (Q19, Q30). For the latter case, the number does not add up to the respective “N,” and the proportion does not add up to 100%. There was an option to provide freeform responses to four questions (Q15, Q21–22, Q32), which is not shown below. We did not group them further and are free to explore in the raw dataset [1]. Similarly, there was an option to provide a free-form response through the “Other” choice for twelve questions (Q4–8, Q11–12, Q16, Q18–20, Q25). We have preprocessed responses to some of these questions, See “Data preprocessing” section.
(DOCX)

S1 File Data preprocessing and summary of survey responses. (DOCX)
