# Supplementary material for: Impact of the COVID-19 pandemic on computational biology early career researchers: A global retrospective study
Source: PLoS Comput Biol. 2025 Oct 15;21(10):e1013554. doi: 10.1371/journal.pcbi.1013554 (PMC12527158; doi:10.1371/journal.pcbi.1013554)
Supplement: S1 Table — Descriptive statistics of responses to the Survey data in 2020 and 2021. There were 284 and 75 responses in the 2020 and 2021 datasets, respectively. If there is a change in the number of responses (N), “N” is marked accordingly next to the question. The questions compulsory to answer are marked with “*” in the first column: Q1–9, Q11–13, Q16–18, Q20–25, and Q27–31. Respondents have the option to select only one choice (Q1–14, Q16–18, Q20, Q23–29, Q31) and, to some questions, have the option to select multiple choices (Q19, Q30). For the latter case, the number does not add up to the respective “N,” and the proportion does not add up to 100%. There was an option to provide freeform responses to four questions (Q15, Q21–22, Q32), which is not shown below. We did not group them further and are free to explore in the raw dataset [1]. Similarly, there was an option to provide a free-form response through the “Other” choice for twelve questions (Q4–8, Q11–12, Q16, Q18–20, Q25). We have preprocessed responses to some of these questions, See “Data preprocessing” section. (DOCX) [file pcbi.1013554.s001.docx]

# Data summary

**S1 Table.** **Descriptive statistics of responses to the Survey data in 2020 and 2021.** There were 284 and 75 responses in the 2020 and 2021 datasets, respectively. If there is a change in the number of responses (N), “N” is marked accordingly next to the question. The questions compulsory to answer are marked with “*” in the first column: Q1-9, Q11-13, Q16-18, Q20-25, and Q27-31. Respondents have the option to select only one choice (Q1-14, Q16-18, Q20, Q23-29, Q31) and, to some questions, have the option to select multiple choices (Q19, Q30). For the latter case, the number does not add up to the respective “N,” and the proportion does not add up to 100%. There was an option to provide freeform responses to four questions (Q15, Q21-22, Q32), which is not shown below. We did not group them further and are free to explore in the raw dataset [[1]](https://www.zotero.org/google-docs/?0TcbAN). Similarly, there was an option to provide a free-form response through the “Other” choice for twelve questions (Q4-8, Q11-12, Q16, Q18-20, Q25). We have preprocessed responses to some of these questions, See “Data preprocessing” section.

| **Q No.** | **Question (Options)** | **Responses in 2020**  (N=284) | **Responses in 2021**  (N=75) |
| --- | --- | --- | --- |
| 1* | What is your gender? |  |  |
|  | Male, n(%) | 153 (53.87) | 34 (45.33) |
|  | Female, n(%) | 126 (44.37) | 38 (50.67) |
|  | Non-binary, n(%) | 4 (1.41) | 3 (4.00) |
|  | Prefer not to say, n(%) | 1 (0.35) | 0 |
| 2* | Please indicate your age range: | N=283 (NA=1) |  |
|  | 18-25, n(%) | 53 (18.73) | 20 (26.67) |
|  | 26-30, n(%) | 111 (39.22) | 21 (28.00) |
|  | 31-35, n(%) | 59 (20.85) | 20 (26.67) |
|  | 36-40, n(%) | 31 (10.95) | 7 (9.33) |
|  | 41-45, n(%) | 15 (5.30) | 2 (2.67) |
|  | 46+, n(%) | 13 (4.59) | 4 (5.33) |
|  | Prefer not to say, n(%) | 1 (0.35) | 1 (1.33) |
| 3* | Where are you geographically located? |  |  |
|  | Asia, n(%) | 27 (9.51) | 14 (18.67) |
|  | Europe, n(%) | 109 (38.38) | 23 (30.67) |
|  | North America, n(%) | 54 (19.01) | 15 (20.00) |
|  | South America, n(%) | 64 (22.54) | 19 (25.33) |
|  | Africa, n(%) | 18 (6.34) | 3 (4.00) |
|  | Antarctica, n(%) | 0 | 0 |
|  | Australia, n(%) | 12 (4.23) | 1 (1.33) |
|  | Prefer not to say, n(%) | 0 | 0 |
| 4* | Please select your current career stage from the list below |  |  |
|  | Undergraduate, n(%) | 24 (8.45) | 8 (10.67) |
|  | Masters, n(%) | 25 (8.80) | 20 (26.67) |
|  | PhD student, n(%) | 115 (40.49) | 21 (28.00) |
|  | PostDoc, n(%) | 48 (16.90) | 10 (13.33) |
|  | Staff Researcher/ Scientist/  Bioinformatician/Consultant, n(%) | 34 (11.97) | 7 (9.33) |
|  | Non-tenured Lecturer/Assistant Professor/  Associate Professor/Group Leader, n(%) | 18 (6.34) | 2 (2.67) |
|  | Tenured Lecturer/Professor/Group Leader, n(%) | 10 (3.52) | 3 (4.00) |
|  | Industry, n(%) | 6 (2.11) | 2 (2.67) |
|  | Other, n(%) | 4 (1.14) | 2 (2.67) |
| 5* | How COVID-19 has impacted the functionality of your institution/organisation? |  |  |
|  | Fully operational | 56 (19.71) | 16 (21.33) |
|  | Partial closure (<50% operational) | 105 (36.97) | 42 (56.00) |
|  | Full closure (<5% operational) | 107 (37.68) | 17 (22.67) |
|  | Other | 16 (5.63) | 0 |
| 6* | What was/is your working situation during lockdown? |  |  |
|  | Work from home policy, n(%) | 241 (84.86) | 47 (62.67) |
|  | Work at the office keeping social distancing measure, n(%) | 8 (2.82) | 2 (2.67) |
|  | A combination of work from home and work at the office, n(%) | 29 (10.21) | 23 (30.67) |
|  | Unable to work from home, n(%) | 6 (2.11) | 3 (4.00) |
| 7* | How would you rate your productivity level (in %) during COVID-19 restrictions compared to normal working conditions? |  |  |
|  | 0-9 | 3 (1.06) | 2 (2.67) |
|  | 10 | 4 (1.41) | 1 (1.33) |
|  | 20 | 8 (2.82) | 2 (2.67) |
|  | 30 | 18 (6.34) | 3 (4.00) |
|  | 40 | 14 (4.93) | 12 (16.00) |
|  | 50 | 26 (9.15) | 10 (13.33) |
|  | 60 | 35 (12.32) | 8 (10.67) |
|  | 70 | 38 (13.38) | 12 (16.00) |
|  | 80 | 47 (16.55) | 7 (9.33) |
|  | 90 | 30 (10.56) | 6 (8.00) |
|  | 100 | 41 (14.44) | 10 (13.33) |
|  | >100 | 11 (3.87) | 1 (1.33) |
|  | Other | 9 (3.17) | 1 (1.33) |
| 8* | Would you suggest your institution/organisation to transition and adopt to work from home approach frequently post- COVID-19? |  |  |
|  | Yes | 172 (60.56) | 46 (61.33) |
|  | No | 38 (13.38) | 13 (17.33) |
|  | Not sure | 74 (26.06) | 15 (20.00) |
|  | Other | 0 | 1 (1.33) |
| 9* | Have you lost any research/work due to the COVID-19 situation? |  |  |
|  | Yes | 87 (30.63) | 28 (37.33) |
|  | No | 197 (69.37) | 47 (62.67) |
| 10 | If you answered yes to question 9, please indicate the worth of work (in months) lost during COVID-19. | N=94 (NA= 190) | N=28 (NA=47) |
|  | < 1 month | 23 (24.47) | 1 (3.57) |
|  | 1-3 months | 47 (50.00) | 11 (39.29) |
|  | 3-6 months | 19 (20.21) | 13 (46.43) |
|  | >6 months | 5 (5.32) | 3 (10.71) |
| 11* | Have you incurred any financial loss in your current project(s) during COVID-19 restricted situation? |  |  |
|  | My own salary is affected | 38 (13.38) | 16 (21.33) |
|  | Lost grant funding ( research/phd grant) in the current cycle | 20 (7.04) | 13 (17.33) |
|  | No economic/financial hurdle incurred | 218 (76.76) | 44 (58.67) |
|  | Other | 8 (2.82) | 2 (2.67) |
| 12* | Did you manage to keep regular communication with your teammates and line manager/supervisor? |  |  |
|  | Yes, more than before the pandemic | 64 (22.54) | 18 (24.00) |
|  | No, less than before the pandemic | 116 (40.85) | 33 (44.00) |
|  | Does not apply | 3 (1.06) | 2 (2.67) |
|  | Same as before | 101 (35.56) | 22 (29.33) |
|  | Other | 0 | 0 |
| 13* | Where is your institution/organisation located? | N=283 (NA=1) |  |
|  | It is in my home country | 215 (75.70) | 57 (76.00) |
|  | It is in a different but nearby country (same continent) | 28 (9.86) | 8 (10.67) |
|  | It is on a different continent | 40 (14.08) | 10 (13.33) |
| 14 | If you are a research student, have you defended your thesis/PhD or taken an exam during lockdown from home? | N=198 (NA=86) | N=52 (NA=23) |
|  | Yes | 15 (7.58) | 17 (32.69) |
|  | No | 147 (74.24) | 25 (48.08) |
|  | It's due in the near future | 36 (18.18) | 10 (19.23) |
| 16* | Do you find teamwork/co-sharing (developing algorithms/writing code/GitHub/Bitbucket) challenging during COVID-19? |  |  |
|  | Yes, more than before the pandemic | 88 (30.99) | 32 (42.67) |
|  | No, less than before the pandemic | 29 (10.21) | 16 (21.33) |
|  | Same as before | 167 (58.80) | 26 (34.67) |
|  | Other | 0 | 1 (1.33) |
| 17* | Rate your current work related stress level (0= no stress, 5 = extremely stressed) |  |  |
|  | 1 | 9 (3.17) | 4 (5.33) |
|  | 2 | 40 (14.08) | 3 (4.00) |
|  | 3 | 101 (35.56) | 27 (36.00) |
|  | 4 | 95 (33.45) | 28 (37.33) |
|  | 5 | 39 (13.73) | 13 (17.33) |
| 18* | Does your institution/organisation have support available for staff/students for mental health and well-being during the COVID-19 pandemic? |  |  |
|  | Yes, n(%) | 152 (53.52) | 30 (40.00) |
|  | No, n(%) | 115 (40.49) | 42 (56.00) |
|  | Other, n(%) |  |  |
|  | Don’t know, n(%) | 17 (5.99) | 3 (4.00) |
| 19 | What type of career development opportunity you are equipped with from your institution/organisation? | N=242 (NA=42) | N=66 (NA=9) |
|  | Career development virtual training | 103 (42.56) | 22 (33.33) |
|  | Training with teammates (e.g. journal club/ knowledge/ virtual hackathon) | 120 (49.59) | 28(42.42) |
|  | Engaging in scientific activity ( e.g. virtual seminar/ webinar / COVID-19 data share/analysis) | 182 (75.21) | 46(69.7) |
|  | Voluntary activity to support affected community | 48 (19.83) | 14 (21.21) |
|  | Other | 7 (2.89) | 3 (4.55) |
| 20* | Which of the following mediums appeals to you personally in terms of working methods during the COVID-19 pandemic? |  |  |
|  | Daily video conferencing (e.g. Team, Zoom, Meetup) in groups | 66 (23.24) | 21 (28.00) |
|  | Daily teleconferencing/video conferencing in groups | 4 (1.41) | 2 (2.67) |
|  | Bi-weekly video conferencing and monthly 1-2-1 with line manager | 53 (18.66) | 18 (24.00) |
|  | Weekly teleconferencing/video conferencing | 151 (53.17) | 33 (44.00) |
|  | Other | 10 (3.52) | 1 (1.33) |
| 23* | Please select what percentage ( %) of your working time do you spend in the dry lab (working with computers) |  |  |
|  | 10% | 5 (1.76) | 0 |
|  | 20% | 4 (1.41) | 2 (2.67) |
|  | 30% | 3 (1.06) | 4 (5.33) |
|  | 40% | 10 (3.52) | 4 (5.33) |
|  | 50% | 13 (4.58) | 4 (5.33) |
|  | more than 50% | 249 (87.68) | 61 (81.33) |
| 24* | Did the COVID-19 confinement get you far from your country of residence? |  |  |
|  | Yes | 34 (11.97) | 11 (14.67) |
|  | No | 250 (88.03) | 64 (85.33) |
| 25* | Please select your living situation as appropriate from the list below : |  |  |
|  | I live alone, n(%) | 55 (19.37) | 15 (20.00) |
|  | I share with relatives, n(%) | 71 (25.00) | 22 (29.33) |
|  | I share with non-relatives, n(%) | 23 (8.10) | 5 (6.67) |
|  | I live with partner without kids, n(%) | 82 (28.87) | 20 (26.67) |
|  | I live with partner with kids, n(%) | 45 (15.85) | 12 (16.00) |
|  | I am a single parent, n(%) | 3 (1.06) | 1 (1.33) |
|  | Other, n(%) | 5 (1.76) | 0 |
| 26 | In case you answered, you have kids under your care. How much time overload have you got because of childcare? | N=57 (NA=227) | N=14 (NA=61) |
|  | 10% | 10 (17.54) | 1 (7.14) |
|  | 20% | 6 (10.53) | 1 (7.14) |
|  | 30% | 4 (7.02) | 3 (21.43) |
|  | 40% | 14 (24.56) | 3 (21.43) |
|  | More than 50% | 23 (40.35) | 6 (42.86) |
| 27* | How much time overload have you got on a daily basis because of domestic tasks than pre-COVID (cooking, cleaning, homeschooling, etc) |  |  |
|  | 10% | 93 (32.75) | 19 (25.33) |
|  | 20% | 75 (26.41) | 17 (22.67) |
|  | 30% | 60 (21.13) | 14 (18.67) |
|  | 40% | 32 (11.27) | 18 (24.00) |
|  | More than 50% | 24 (8.45) | 7 (9.33) |
| 28* | Do you have a proper space in your residence for working? |  |  |
|  | Yes, I do have a dedicated space for work | 152 (53.52) | 28 (37.33) |
|  | No, I work in a shared space (living room/kitchen/bedroom) | 132 (46.48) | 47 (62.67) |
| 29* | Do you have a reasonable internet connection suitable for your work needs? |  |  |
|  | Yes | 245 (86.27) | 63 (84.00) |
|  | No | 39 (13.73) | 12 (16.00) |
| 30* | Do you have all the equipment you need for doing a home office? Choose from below ( You can choose multiple options for this question): |  |  |
|  | Proper Laptop/Desktop Computer | 269 (94.72) | 71 (94367) |
|  | Proper Chair | 143 (50.35) | 36 (48) |
|  | Proper Desktop (Table) | 152 (53.35) | 38 (50.67) |
|  | Screen | 169 (59.51) | 45 (60) |
|  | Conference devices (camera/microphone/headphones) | 188 (66.2) | 49 (65.33) |
|  | Mouse | 206 (72.54) | 48 (64) |
| 31* | Did you receive financial help to acquire any needed equipment during work from home time? |  |  |
|  | No, I did not get any equipment, n(%) | 150 (52.82) | 39 (52.00) |
|  | I did not/ do not need any equipment, n(%) | 43 (15.14) | 3 (4.00) |
|  | Yes, I got financial help from my institution to buy needed equipment(s), n(%) | 19 (6.69) | 4 (5.33) |
|  | I got new equipment from my own project/grant/resource funding, n(%) | 7 (2.46) | 4 (5.33) |
|  | Partial support provided to buy/acquire the equipment(s) needed, n(%) | 7 (2.46) | 8 (10.67) |
|  | I needed equipment but there was no financial/resources facility available to provide me by my institution, n(%) | 11 (3.87) | 8 (10.67) |
|  | I got new equipment from my personal salary, n(%) | 47 (16.55) | 9 (12.00) |
