# Supplementary material for: Impact of the COVID-19 pandemic on computational biology early career researchers: A global retrospective study
Source: PLoS Comput Biol. 2025 Oct 15;21(10):e1013554. doi: 10.1371/journal.pcbi.1013554 (PMC12527158; doi:10.1371/journal.pcbi.1013554)
Supplement: S1 File — (DOCX) [file pcbi.1013554.s002.docx]

**S1 File**

*Eranti, Hegde et al., (2024) Impact of the COVID-19 Pandemic on Computational Biology Early Career Researchers: A Global Retrospective Study.*

# Data preprocessing

The raw data used in this study are available in the Zenodo repository [[1]](https://www.zotero.org/google-docs/?pifHoX). The data preprocessing was performed separately, in both datasets, for each question as and when required, as mentioned below.

## **2020 dataset**

● Question 2 asked participants to select their age range from the following options: 18-25, 26-30, 31-35, 36-40, 41-45, 46-50, 51-55, 56-60, 61-65, and 65+. One individual provided a textual answer of ‘46+’, so it was not possible to determine their exact age range. Furthermore, only 12 participants answered that their age range was above 45 (46-50, 51-55, etc), so there were too few responses in each of these categories to draw meaningful conclusions about these groups. To address these two issues, participants who answered that their age was above 45 were grouped into a single category of ‘46+’.

● Question 4 asked participants to select their current career stage from several academic career stages or a separate category called “Industry.” Four participants provided the following responses: “Non-associated,” “Project Manager,” “Graduate,” and “back to uni.” As these responses did not fit neatly into any of the established categories, they were categorized as “Other”.

● Question 6 asked participants to categorize their work situation during lockdown into one of the following: “Work from home policy,” “Work at office keeping social distancing measures,” “A combination of work from home and work at office,” and “Unable to work from home.” One participant provided a text response of “Home + office + wetlab,” which was put into the “A combination of work from home and work from office” category.

● Question 14 asked participants who were research students whether they had defended their thesis or taken an exam remotely during lockdown. One participant marked both “Yes'' and “No”, presumably by accident. Upon investigation, this response was provided by a postdoctoral researcher, i.e., not a research student. Hence, the response was substituted with “N/A''.

● Question 16 asked participants whether they found teamwork and co-sharing more difficult, less difficult, or the same compared to before the pandemic. Four participants provided longer-form text responses. Upon analysis, these responses all fit into the “Same as before” category and were substituted as such.

## **2021 dataset**

● For question 6, the majority of participants provided answers within the existing categories. One participant answered, “I work in the hospital Pesquisa Neonatal”; this was put into the “Unable to work from home” category.

● Question 7 asked participants how they felt their productivity level during the pandemic differed compared to that before the pandemic. One participant answered “120”, which was re-categorized as “>100%”.

**References**

[1. Eranti P, Hegde M, Al Sium SM, Parra RG, Kilpatrick AM, Shome S, et al. Impact of the COVID-19 pandemic on computational biology ECRs: survey data 2020 and 2021. Zenodo; 2023. doi:10.5281/ZENODO.8427807](https://www.zotero.org/google-docs/?g08fK3)
